# Ecological Analysis of the Helminth Community of the Wood Mouse, *Apodemus sylvaticus*, along an 18-Year Post-Fire Regeneration Period in a Mediterranean Ecosystem

**DOI:** 10.3390/ani11102926

**Published:** 2021-10-10

**Authors:** Sandra Sáez-Durán, Ángela L. Debenedetti, Sandra Sainz-Elipe, Mireia Sabater-Tena, María Teresa Galán-Puchades, Màrius Vicent Fuentes

**Affiliations:** Parasites & Health Research Group, Departament de Farmàcia i Tecnologia Farmacèutica i Parasitologia, Facultat de Farmàcia, Universitat de València, Av. Vicent Andrés Estellés s/n, Burjassot, 46100 València, Spain; sandra.saez@uv.es (S.S.-D.); angela.debenedetti@uv.es (Á.L.D.); sandra.sainz@uv.es (S.S.-E.); misate@alumni.uv.es (M.S.-T.); mteresa.galan@uv.es (M.T.G.-P.)

**Keywords:** helminth community, wood mouse, *Apodemus sylvaticus*, post-fire, regeneration process, Mediterranean ecosystem, Serra Calderona, Spain

## Abstract

**Simple Summary:**

After a wildfire, especially in Mediterranean ecosystems, the vegetation starts to recover gradually but appears regenerated after a decade. However, the study of helminth parasites of the wood mouse, the most important post-fire recolonizer mammal in the western Mediterranean forests, allowed us to elucidate the reality of this process more accurately, mainly due to the, sometimes complex, life cycles of the helminths using other hosts (vertebrates and invertebrates) to complete them. Thus, the comparative analysis of various aspects (biodiversity and kinds of life cycles) of the helminth community of the wood mouse, as well as the influence of some external factors (related to the environment and its periodical post-fire regeneration) and internal factors (related to the population of the wood mouse), between the burned area and a non-burned area used as control, reveals the biological indicators (indicators of the situation/evolution of a process) of the post-fire regeneration process and its true state. The current study confirms the important role of the helminths of the wood mouse as biological indicators and the influence that climate variables exercise on the quality and evolution of the post-fire regeneration process in Mediterranean ecosystems.

**Abstract:**

The role of helminths of the wood mouse, *Apodemus sylvaticus*, as biological indicators of the post-fire regeneration process in Serra Calderona Natural Park, a Mediterranean forest ecosystem located between the provinces of València and Castelló (Valencian Country, Spain), has been analysed for almost twenty years. The helminth ecological analysis of 917 *A. sylvaticus* (675 originating from the burned area and 242 originating from the control area) has been carried out between the 2nd and 18th post-fire years. The influence of intrinsic (host population density, sex and age) and extrinsic (site, period and year of capture, climate variables) factors on the post-fire evolution of the helminth community of the wood mouse, and the biodiversity, species richness and life cycle of the helminth species was studied. Taking into account the most important results obtained, various aspects of the helminth community dynamics of the wood mouse are confirmed as biological indicators of the post-fire regeneration process in Mediterranean ecosystems. The still existing differences between the two areas are mainly related to the influence of climate variables on the post-fire regeneration process. Moreover, the important role that helminth parasites of the wood mouse play as biological indicators of this process in Mediterranean ecosystems is demonstrated.

## 1. Introduction

The close relationship between parasites and their hosts has made it possible to propose parasites as biological tags of biological aspects, population dynamics and even of the phylogeny of their hosts [1,2,3,4] as well as biological indicators of the impact of environmental disasters, whether they be aquatic or terrestrial [5,6,7,8,9,10,11,12,13,14,15,16,17,18].

With regard to the contribution of parasites to the ecology and the interactions of their host with the ecosystem, based on the trophic transmission of numerous parasite species, the observations of Marcogliese [19] stand out. The knowledge of the life cycle of parasites, especially those with an indirect heteroxenous cycle, is relevant because it can be related with the composition of the host diet. In the case of small mammals, the importance of the ingestion of invertebrates by a host species could be elucidated from the composition and the structure of its helminth community, both in species with mainly an insectivorous diet as well as those with a granivorous diet. The presence of certain parasite species is able to provide valid information about the fauna of invertebrates present in the study area, especially when the specificity of a parasite at its intermediate host level is either oioxenous (the species level) or stenoxenous (the genus level). Moreover, those helminth species with a heteroxenous cycle that use small mammals as intermediate or paratenic hosts, can reveal information about the presence of the definitive hosts, mainly carnivorous and raptors. On the other hand, helminth species with a direct or monoxenous cycle can provide information on the environmental conditions of the study area as the presence and the viability of certain resistant stages of the parasite will be favoured or biased depending on the climatology and seasonal variability.

In Europe, helminth parasites of sylvatic rodents have been the subject of numerous studies, both at a continental as well as an insular level. Some of these studies are merely limited to the report of the helminth species present in certain rodent host species. However, other studies have analysed the ecological aspects that influence the helminth/host relationship in depth. These studies were mainly carried out in central and eastern Europe, Finland and the British Isles [20,21,22,23,24,25,26,27,28,29,30,31], as well as several Mediterranean insular enclaves [32,33,34]. In the Iberian Peninsula, relevant studies were carried out in: Albufera of València [35], Serra da Malcata, Portugal [36], Doñana National Park [37], Serra Calderona Natural Park (NP), Castelló-València [38], Sierra de Espuña, Murcia [39], Sierra de Gredos, Ávila [40], Dunas de Mira, Portugal [41], and the Erro river valley, Navarre [42].

Parasites do not only have the capacity to regulate the host population, they also provide information on possible changes in the behaviour of their hosts, as mathematically demonstrated by the theories of Anderson and May [43,44,45,46,47,48]. Theories that have been subsequently backed up on numerous occasions by field studies, mainly those carried out using annual series and comparing vulnerable areas affected by an environmental disaster to stable control areas [13,14,16,17,18].

Wildfires have had the most devastating and frequent impact on Mediterranean ecosystems throughout the last 150 years, affecting the stability of these forest ecosystems together with the soil, vegetation and fauna [49,50,51,52], given that they influence soil preservation and the recovery of the vegetation and fauna.

The forest regeneration process and the evolution of the ecosystem take time and are always impacted by the environmental conditions, especially the climate, which can be evaluated considering the quality of soil preservation and the recovery of the vegetation and fauna [50,51,53,54].

The impact of a wildfire on terrestrial vertebrates produces, as in the case of invertebrates, a high degree of mortality. However, after the wildfire those animals that fled will return, and those who died will be replaced by others of the same or different species. This process is the beginning of the animal recolonisation, which takes place parallel to the regeneration of the forest vegetation. Numerous studies carried out in various Mediterranean forest ecosystems, mostly in Western Europe (among others: [53,55,56,57,58,59,60,61,62,63]) but also in other Mediterranean forest enclaves, such as in Australia, concluded that small mammals become, by means of their recolonisation process, the most valuable biological indicators of the forest regeneration. In the European Mediterranean ecosystems, the wood mouse, *A. sylvaticus*, a generalist rodent whose diet is mainly based on grains and insects, has shown itself to be the most important and efficient post-fire recolonizer [53].

However, this recognised role of small mammals as biological indicators is limited in time, subsequently being replaced by their helminth parasites (trematodes, cestodes, nematodes and acantocephalans) as biological indicators of the overall regeneration of the ecosystem as they are bioecologically more demanding. Consequently, the study of helminth parasites reveals, at any given moment, the state and the tendencies of the post-fire regeneration process [6,9,10].

There are only a few studies of the use of helminths of small mammals as biological indicators of the post-fire regeneration process in Mediterranean forest enclaves, and most of them, with the exception of that of Spratt in Australia [5], were undertaken in the east and the northeast of the Iberian Peninsula and the eastern Pyrenees [9,64,65]. Other research carried out, for example in Montseny NP [66], was not long term.

After the wildfire of 1992 in Serra Calderona NP, a multidisciplinary project concerning the role of helminths of small mammals as biological indicators of the post-fire regeneration process was initiated. Hitherto, various periods have been analysed. The results of which have shown the trends of the helminth community of the wood mouse, *A. sylvaticus*, from a bioecological point of view, between the 2nd and 5th post-fire year (PFY) [14] and between the 2nd and 10th PFY [17], as well as from the component species point of view during the 2nd to 10th PFY period [67]. Other mammals, such as the common shrew, *C. russula*, were analysed from the component species point of view during the 2nd to 11th PFY period [13], and the Mediterranean mouse, *M. spretus*, during the 2nd to 14th PFY period [18].

The main objective of the present study is to verify the role of the helminths of the wood mouse as biological indicators of the post-fire regeneration process in Serra Calderona NP during the 2nd to 18th PFY period. The specific objectives are: to characterize the composition and structure of the helminth community of the wood mouse in this Mediterranean enclave; to analyse the annual changes in the helminth community of the wood mouse in the burned area, in relation to the nature of the biological cycles of the helminth species and the helminth community richness and diversity as well as the influence of intrinsic and extrinsic factors.

## 2. Materials and Methods

**Figure 1:**
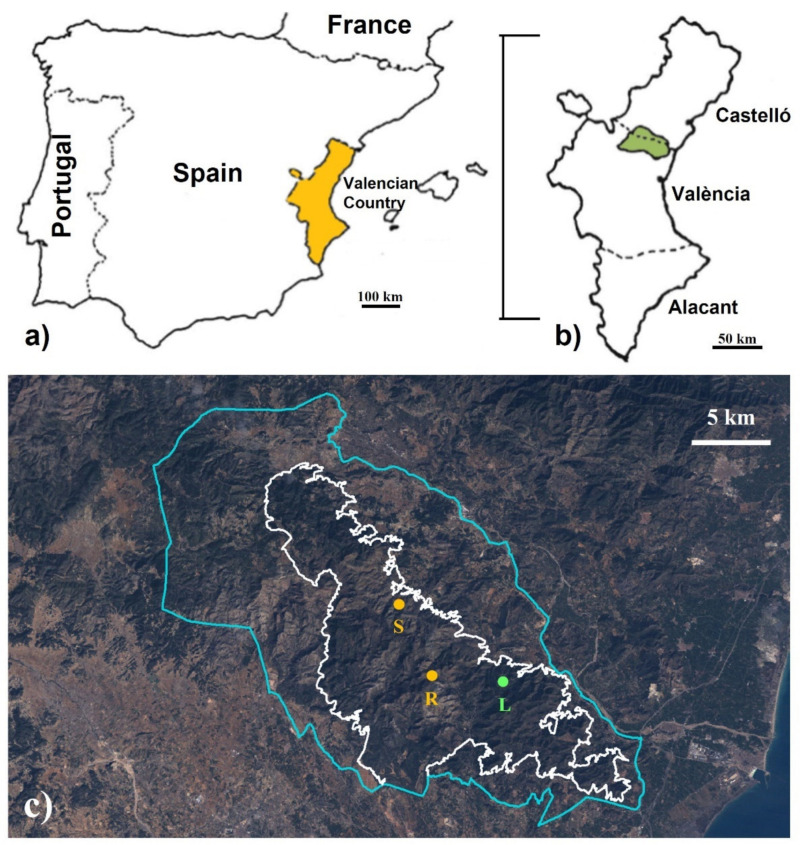
Study area: (**a**) location of Valencian Country in the Western Mediterranean frame; (**b**) location of Serra Calderona Natural Park in the Valencian Country; (**c**) Landsat 5 TM image of February 1994 (courtesy of the U.S. Geological Survey) showing the location of the three trapping sites in the burned (S—La Saladilla; R—Rebalsadors) and in the control (L—Les Llomes) areas in Serra Calderona Natural Park (blue outline) and in its special protection area (white outline).

The study area, the Serra Calderona NP, is a Mediterranean ecosystem located between the provinces of Castelló and València in the Valencian Country, Spain (39°35′–39°51′ N, 0°15′–0°43′ W) (Figure 1). This mountain range, which covers approximately 52,000 ha of forests and cultivated land untended, suffered a large wildfire at the end of the summer of 1992 devastating a total of 9500 ha.

The Serra Calderona NP has a typical Mediterranean climate, with irregular rainfall and intense summer droughts. The eastern and meridional regions correspond to the thermomediterranean bioclimatic setting with a dry ombroclimate. In contrast, the westernmost and septentrional regions are included in the mesomediterranean bioclimate, with a little more rainfall but not fully qualifying as a subhumid ombroclimate [68]. Chorologically, the vegetation corresponds to the Western Mediterranean sub-region [69,70]. As a consequence of the continuous degradation due to anthropization (growing of crops, charcoal production, animal grazing and building activities), as well as numerous wildfires, potential vegetation is largely restricted to various substitution stages, principally littoral kermes oak (*Quercus cocciferae–Pistacietum lentisci*) and continental kermes oak (*Rhamno lycioidis–Quercetum cocciferae*), together with different types of shrubs and pastures.

In February 1994, during the winter of the 2nd PFY, a multidisciplinary project concerning the post-fire recolonization dynamics of small mammals, mainly the wood mouse, *A. sylvaticus*, and the study of their helminth parasites as biological indicators in the process of post-fire regeneration, was initiated.

### 2.1. Zoological and Helminthological Procedures

**Figure 2:**
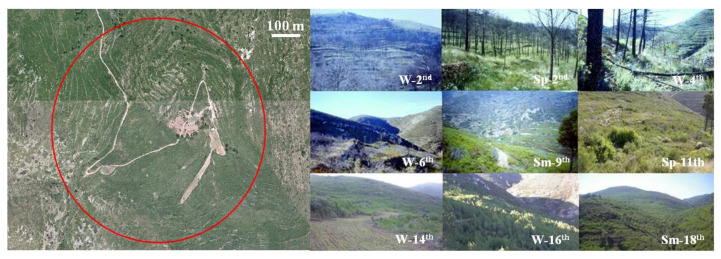
Orthophoto (courtesy of the Valencian Cartographic Institute) of the trapping site of La Saladilla (burned area) and some photographs showing its evolution during the post-fire regeneration process (W = Winter; Sp = Spring; Sm = Summer).

**Figure 3:**
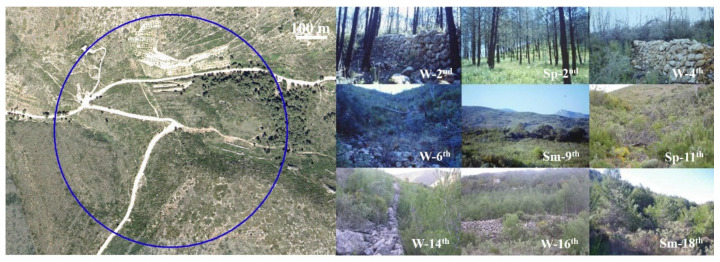
Orthophoto (courtesy of the Valencian Cartographic Institute) of the trapping site of Rebalsadors (burned area) and some photographs showing its evolution during the post-fire regeneration process (W = Winter; Sp = Spring; Sm = Summer).

**Figure 4:**
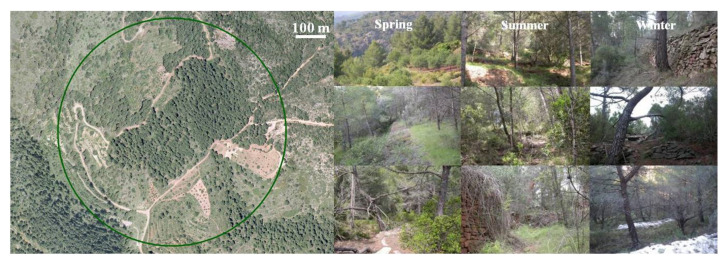
Orthophoto (courtesy of the Valencian Cartographic Institute) of the trapping site of Les Llomes (control area) and some seasonal photographs taken during the study period.

**Figure 5:**
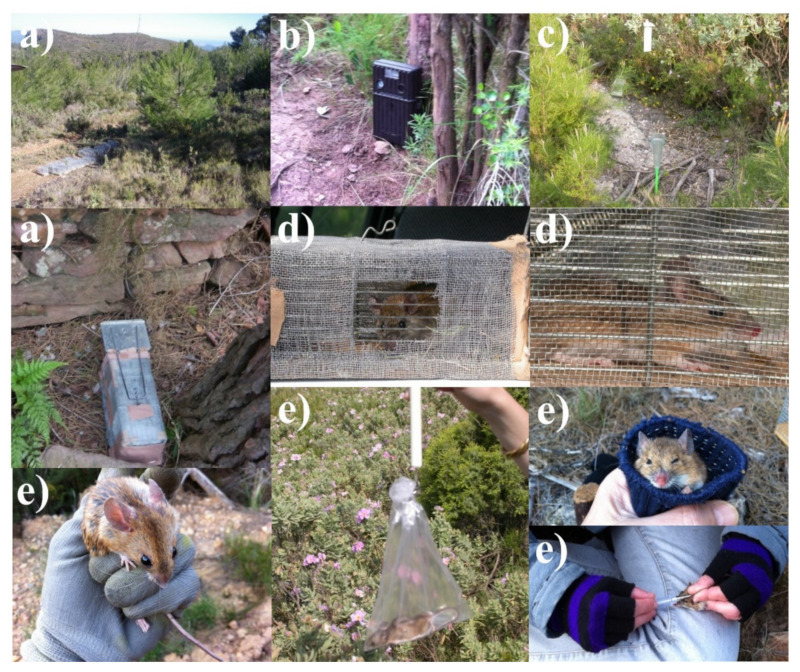
Some snapshots showing the most common tasks during surveys: trap preparation (**a**), camera trapping (**b**), temperature and rainfall measurement (**c**), captures (**d**), handling and marking of small mammals captured (**e**).

Three trapping sites were set up within the study area for annual–seasonal follow-ups, two in the burned area (Figure 2 and Figure 3) and one in the control area (Figure 4). The control area presents the same ecological conditions as the burned area before the fire and is situated in a straight line about 6 km away from the affected area in order to minimize nearness effects resulting from the mobility of the small mammals. The two burned sites are considered together as the burned area as both sites are very close and have a great ecological similarity, minimizing, moreover, the negative effect of the sampling method for the helminthological study. A comprehensive description of the trapping method and study procedures was previously reported [10,38,53]. The trapping method used to capture small mammals is based on the square plot (quadrate) technique using hand-made wire-mesh traps and the capture–release method. On a seasonal basis, at intervals of three to four months, 55 traps were placed at night within a distance of 10–15 m forming a square in each of the three study sites. The trapping took place during three consecutive days checking each trap every 24 hours, and to minimise the influence of the weather on the trapping success. Trapping sessions (all three days) were postponed on days with heavy rainfall or extreme temperatures predicted. The captured animals were identified at species level and weighed, sexual activity was determined (testicular descent in males, and vulvar opening, pregnant and lactating conditions in females were checked), they were marked with a lasting colorant on different parts of the abdomen and/or the thorax and a subcutaneous microchip was introduced using an individual disposable syringe (Avid® Inc.). The animals were then released at the place of capture [71] (Figure 5).

The wood mouse population density in each area (burned and control) was determined by the number of individuals captured per 100 trap-nights, and was analysed to determine possible differences due to the post-fire regeneration process [71].

During the study period, from the 2nd (1994) to the 18th (2010) PFY, 217 prospections were carried out with a total of 29,011 traps and 3353 small mammals captured, i.e., with a success rate of 11.56%. Animals captured were: 2791 wood mice, *Apodemus sylvaticus*, (9.62%); 283 Mediterranean mice, *Mus spretus*, (0.98%); 25 black rats, *Rattus rattus*, (0.09%); 11 garden dormice, *Eliomys quercinus*, (0.04%); 243 greater white-toothed shrews, *Crocidura russula*, (0.84%).

Dead individuals and, between the period from the 2nd to the 5th PFY only, fewer than 10% of specimens captured alive that were euthanized by exposure to CO_2_-saturated atmosphere, were dissected and used for the helminthological study. As previously described [14,38], all helminths were collected and preserved. Trematodes and cestodes were preserved in 70% ethanol, stained with Grenacher’s boracic and alcoholic chlorhydric carmine, respectively, differentiated with acidified ethanol, dehydrated in an alcohol series, cleared with xylene and mounted in Canada balsam. Nematodes were preserved in ethanol 70% and cleared in Amann lactophenol. All helminths were identified at specific level based on their morphology and morphometry and according to the most relevant descriptions and findings of Trematoda [72], Cestoda [73,74,75,76,77,78,79,80] and Nematoda [81,82,83,84,85,86,87,88,89,90,91,92,93]. However, a number of specimens could not be specifically classified due to their limited development.

Herein, the helminthoecological analysis of 917 individual wood mice, *A. sylvaticus*, captured prior to September 2010, the summer of the eighteenth PFY, is included. Six hundred and seventy five individual hosts originated from the burned area and 242 from the control area. The number of *A. sylvaticus* captured in each PFY, as well as their annual population density, expressed by the number of individuals captured per 100 trap-nights for the burned and the control areas are summarized in Table 1.

### 2.2. Helminth Community Analysis

A global comparison of the burned and the control area was conducted. The analysis of the helminth community composition and structure for both areas was carried out considering each particular life cycle and calculating the prevalence, mean abundance, median intensity and range [94]. Where possible, standard non-parametric tests were applied [95].

As proposed by Fuentes et al. [14,96], two different types of life cycles for helminths were considered: helminths classified as FES, which have a free-environmental infectious stage for the wood mouse, and helminths classified as no-FES, invertebrate-borne helminths, which use at least one invertebrate as intermediate host. Moreover, nematode species with a direct or monoxenous life cycle were also classified into three different groups, i.e., ageohelminths, pseudogeohelminths and geohelminths. Ageohelminths include those nematodes that release embryonated and directly infective eggs, or that embryonate in a short period of less than four hours, such as species of the genus *Syphacia*. Pseudogeohelminths are nematodes that release non-embryonated eggs, which require a development period usually of 2–3 weeks depending on the climate, in the environment to embryonate and, consequently, be infective, such as species of the genus *Trichuris*. Geohelminths are those nematodes that have a free-living larval stage in the environment as the infective form, such as species of the genus *Heligmosomoides*.

The analysis of the helminth community components was carried out by means of calculating the frequency of occurrence of the number of helminth species, which refers to the infracommunities of the host, i.e., to the number of helminth species present in each host individual and expressed as a percentage, showing the distribution of the helminth community in the host population [14,17].

Helminth community diversity describes the composition of a community in terms of the number of species present and some factor that weighs the relative evenness of distribution of each species [94]. Its analysis was completed using the following indices: Shannon index (*H*) [97,98]; Simpson index (*D*), expressed as 1-*D* [98,99]; Berger–Parker index (*d*), expressed as 1-*d* [98,100,101]; Shannon evenness (*E*) [98,102].

The helminth infracommunity structure was established through the analysis of the species richness (considering the number of helminth species present in each infracommunity and expressed as the mean of helminth species present in each host population), the Brillouin index (*HB*) [97,98], Brillouin index for infected hosts only and percentage of infected hosts.

The role played by intrinsic factors (host age and sex, and host population density) and extrinsic factors (site, season and year of capture, and five climate variables) (Table 2) in determining the species richness (expressed through the number of helminth species), and the helminth community diversity (expressed by the five diversity indices) of the wood mouse, as well as the prevalence of the different types of life cycles was analysed.

Data of climate variables were obtained from six weather stations of AEMET (Spanish Meteorological Agency) located in the burned and in the control areas. Annual data, average or accumulated values, related to temperature and precipitation, respectively, were obtained from monthly data of the three stations located in each study area (Table 3).

As seasonal effects are strongly related to each PFY, the period of capture (i.e. season of capture linked to the year of capture) was considered as an independent variable. Additionally, the analysis was carried out individually for each area to allow for the consideration of the effect of the fire and the regeneration process from another point of view, i.e., showing the influence of intrinsic and extrinsic factors on each area separately.

Climate data and host population density, values belonging to the year before capture, were correlated with the annual values of the prevalence of different kind of life cycles and values of species richness and the five diversity indices by means of the Spearman correlation coefficient (Rho); prevalence was previously transformed logarithmically, log (X/1 - X), being X the centesimal expression of the percentages.

The influence of the other intrinsic and extrinsic factors, different than climate variables and host density, on the dependent variables, was carried out using a binary logistic regression (BLR) in the case of prevalence of the different kind of life cycles, and using a multifactorial general linear model (specifically ANOVA) in the case of species richness and Brillouin index. A t-test was used to compare statistical differences between the PFY values of Shannon, Simpson, and Berger–Parker indices, as well as Shannon evenness, in both areas.

Statistical analyses were carried out using StatView 5.0 (SAS Institute Inc) and IBM SPSS Statistics 26 (IBM Corporation) for Windows software packages. Statistical significance was established at *p* < 0.05.

## 3. Results

### 3.1. Helminth Community Analysis

**Figure 6:**
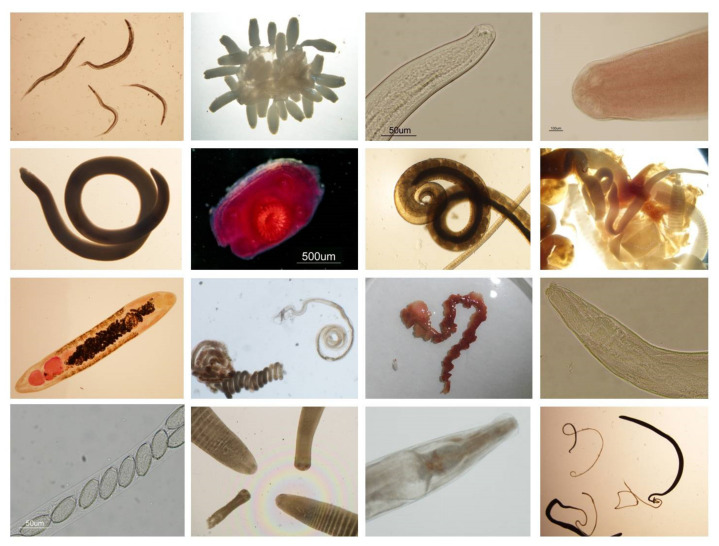
Microscopy photographs of some helminths found in the wood mice analysed. From left to right: 1st line, *Syphacia frederici* females, *Taenia parva* metacestode, anterior part of *Eucoleus bacillatus*, *Pseudocatenotaenia matovi* scolices; 2nd line, *Mastophorus muris* female, *Taenia parva* scolex section, caudal end of *Trichruis muris* male, *Gallegoides arfaai* strobila; 3rd line, *Brachylaima* sp. adult, *Heligmosomoides polygyrus* female and male, *Taenia martis* metacestode, anterior end of *Syphacia frederici* female; 4th line, eggs in the uterus of an *Aonchotheca annulosa* female, *Gallegoides arfaai* and Catenotaeniidae Gen. sp. scolices, anterior end of *Aspiculuris tetraptera* female, *Trichuris muris* females.

The helminthological analysis of the 917 wood mice captured in Serra Calderona NP revealed that the helminth community of the wood mouse in this enclave is composed of a total of sixteen helminth species (Figure 6) and some unidentified nematode larvae, with a total helminth parasitation of 85.06%. Selected characteristics of these species, such as the site of helminth parasitation in the wood mouse, their type of life cycle and other relevant information related to it, are shown in Table 4. The cestode species *Hymenolepis straminea* and *Gallegoides arfaai* were detected in the burned area only.

Total helminth parasitation was 87.70% in the burned and 77.69% in the control area; this difference being statistically significant (*χ*^2^ = 13.291; *P* = 0.0003; *df* = 1).

Ten of the 16 helminth species are considered helminth component species, with a prevalence above 10% in most post-fire years studied: the larva of the cestode *Taenia parva*, found in the abdominal cavity; the intestinal catenotaenid cestodes *Pseudocatenotaenia matovi* and *Skrjabinotaenia lobata*; the pseudogeohelminth nematodes *Trichuris muris* and *Eucoleus bacillatus*; the geohelminth nematode *Heligmosomoides polygyrus*; the ageohelminth nematodes *Syphacia stroma* and *Syphacia frederici*; the diheteroxenous nematodes *Aonchotheca annulosa* and *Mastophorus muris*. Among these helminth component species, *Syphacia stroma* was the most prevalent and abundant species, while *T. parva* was the least prevalent and abundant in the burned area; *H. polygyrus* was the most prevalent and *S. frederici* was the most abundant species, while *S. lobata* was the least in the control area (Table 5).

Concerning the prevalence of the five types of life cycles considered in the study, the higher prevalence of all of them in the burned area, with the only exception being geohelminth nematodes that are more prevalent in the control area, stands out, with all of these differences being statistically significant (Table 6).

**Figure 7:**
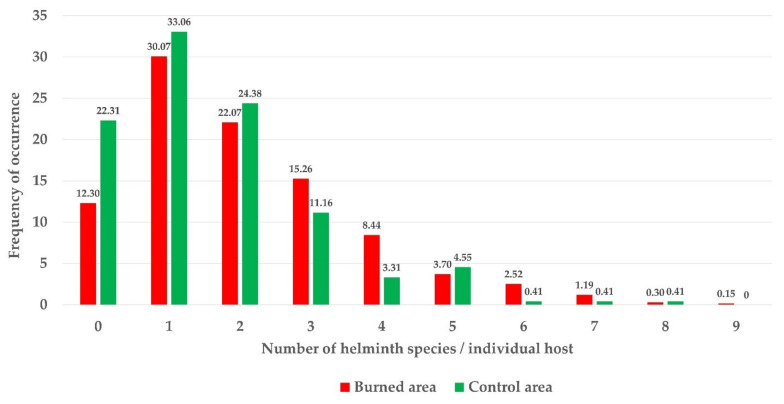
Frequency of occurrence of the number of helminth species present in the infracommunities of the wood mouse in the burned and control areas.

More than 50% of parasitized hosts have helminth infracommunities composed of one or two helminth species in both areas, reaching infracommunities of up to nine species in one individual wood mouse only, captured in the burned area (Figure 7). Moreover, the frequency of the number of helminth species corresponds, in both areas, to a random or Poisson distribution.

The values of the Shannon, Simpson, Berger–Parker and Shannon evenness indices (Table 7) reflect the diversity/uniformity of the helminth community in both areas. The slightly higher values of the three diversity indices in the burned area when compared to the control area reflect that the helminth community in the area in regeneration is somewhat more diverse. However, there were no significant statistical differences between the annual values of these indices in both areas.

Table 8 shows the diversity of the infracommunities of the burned and control areas determined by species richness and the Brillouin index values, including uninfected animals as well as values for infected hosts alone, revealing an almost similar biodiversity in both areas but with higher values in the area affected by the wildfire.

### 3.2. Influence of Intrinsic and Extrinsic Factors

Some correlations between the wood mouse population density and climate variables with diversity, species richness and the kind of life cycles of the helminth community of the wood mouse were found in both areas (Table 9). In the burned area, the prevalence of FES cycles, ageohelminth and pseudogeohelminth nematode species were positively correlated with the number of rainy days, and the species richness with the mean temperature and the number of rainy days. The absence of correlations concerning the prevalence of no-FES cycles and the five diversity indices in the burned area stands out. In the control area, the prevalence of no-FES cycles was negatively correlated with the population density of the wood mouse and precipitation, while the prevalence of FES and geohelminth cycles were negatively correlated with minimum temperature. The fact that all correlations found in the control area were negative and the absence of correlations regarding ageohelminth and pseudogeohelminth nematodes as well as species richness and the five diversity indices is also noteworthy.

The models generated by BLR related to the influence of the period and year of capture, and sex and host age on the prevalence of the five types of cycles are shown in Table 10. In the burned area, the post-fire year of capture influenced the prevalence of nematodes with a geohelminth life cycle; the period of capture influenced the prevalence of helminths with an FES, no-FES, ageohelminth and pseudogeohelminth life cycle; the age of the wood mouse influenced the prevalence of helminths with a no-FES, pseudogeohelminth and geohelminth life cycle. In the control area, the post-fire year of capture influenced the prevalence of helminths with an FES, pseudogeohelminth and geohelminth life cycle; the period of capture influenced the prevalence of helminths with a no-FES and ageohelminth life cycle; the age of the wood mouse influenced the prevalence of helminths with a no-FES, pseudogeohelminth and geohelminth life cycle. The differences found between both areas show the stronger influence of the post-fire period on the helminth community of the burned area. Moreover, the sex of the wood mouse was not significantly related to any of the life cycles considered in the study in both areas.

The influence of these same intrinsic and extrinsic factors on the biodiversity of the helminth community in both areas is shown in Table 11. In the burned area, the post-fire year and the period of capture, as well as the host age, exercised an influence on the species richness and on the values of the Brillouin index; the post-fire year linked to the sex of the host on the species richness; the post-fire period linked to the sex of the host on the species richness and the values of the Brillouin index; the post-fire period linked to the age of the host on the species richness. In the control area, the post-fire year and the host age exercised an influence on the species richness and on the values of the Brillouin index; the post-fire period and the sex linked to the age of the host on the values of the Brillouin index. The influence of the site of capture shows that the species richness in the burned area is highly influenced by the post-fire period, while the values of the Brillouin index, although being different, are influenced by almost the same factors in both areas. Once again, there was no interaction between the sex of the wood mouse and the biodiversity of the helminth community in both areas.

## 4. Discussion

### 4.1. General Analysis

The helminth community of *A. sylvaticus* in Serra Calderona NP, after the helminthological analysis of a total of 917 individuals, is relatively similar to that previously reported in the entirety of the Iberian Peninsula [103]. Of the 28 species reported in that review, 14 of them have also been detected in Serra Calderona. Above all, the absence of trematodes with an aquatic life cycle, the terrestrial trematode *Corrigia vitta*, in spite of the presence of its two intermediate hosts, the nematodes *Rictularia proni*, *Angiostrongylus dujardini* and some capillarin species stands out. These absences can be explained through the limitation of resources in this Mediterranean enclave in comparison with the entirety of the Iberian Peninsula and, specifically, of some of the analysed enclaves that have a greater ecological diversity. However, the larval stages of the cestodes *Taenia martis* and *Mesocestoides* spp. were detected for the first time in this enclave as parasites of a murid species and of the wood mouse in the Iberian Peninsula, respectively [38].

In the burned area, the global prevalence of parasitation (88%) is still higher than in the control area (78%), as reported for previous regeneration periods of this enclave [14,16,17,67]. The presence of the cestodes *H. straminea* and *G. arfaai* in the burned area stands out, although the hymenolepidid was found at the beginning of the study and in two mice only, and the anoplocephalan was not detected until the 6th PFY.

The most prevalent and abundant species in the burned area, *S. stroma*, was reported as such during the 2nd–10th PFY period [17], although, during the 2nd–5th PFY period, *S. frederici* was the most abundant species [14]. In the control area, the highest *H. polygyrus* prevalence was reported during the 2nd–10th PFY period [17,67] and the 2nd–12th PFY period [16], while in the 2nd–5th PFY period [14] it was *S. frederici*. This last species was also the most abundant in the entire study period.

### 4.2. Life Cycles of Helminth

#### 4.2.1. FES and no-FES Cycles

In both areas, the higher parasitation of helminth species with an FES cycle compared with those with a no-FES cycle was observed. The moderate climate and the absence of some invertebrates during some seasons enable the dominance of helminth species that do not depend on intermediate hosts, as corroborated by the observed higher annual fluctuation of the no-FES cycle helminths prevalence. Likewise, the higher global prevalence of parasitation observed in the burned area, with respect to the control area, is also related to a higher presence of both, helminth species with FES and those with no-FES cycles in the area in regeneration. Several studies on the wood mouse reported the same trend in other enclaves of the Iberian Peninsula [36,39,40,42]. Moreover, other studies carried out in Serra Calderona NP, corresponding to previous periods analysed, also showed similar results for the periods 2nd–5th, 2nd–10th and 2nd–12th PFY, respectively [14,16,17].

The positive influence of climate variables, such as the number of rainy days in the year before capture, on the FES cycles in the burned area as well as the negative influence of precipitation and the population density on the no-FES cycles in the control area do not seem to have a convincing explanation. In this sense, Arneberg suggested population density to be a poor indicator of the species richness of indirect life cycle parasites [104]. However, the negative influence of the minimum temperature of the previous year on the FES cycles in the control area could be an indicator of the negative influence of low temperatures on the free environmental stages.

The extrinsic factor post-fire period (PFP) and the intrinsic factor age are determinants of the no-FES cycles, both in the burned and in the control area, probably due to the seasonality of invertebrates and the diet of the wood mouse according to its age subpopulation.

The influence of the PFP on the FES cycles in the burned area, with respect to the influence of the PFY in the control area, is indicative of a strong influence of the season of capture in the area in regeneration. Moreover, between five and ten years after a wildfire, the differences found between the burned and the control areas with regard to the percentage of species with FES cycles, suggest that the changes in this variable may be related to the post-fire regeneration process [14,17]; the host aggregation, in this case in the burned area, favours the transmission of species with a simple life cycle, basically those most commonly transmitted in a direct manner [104,105].

On the other hand, the absence of important influences of the host population density on helminth species with an FES cycle in both areas stands out; being contrary to the conclusions reached concerning the central role population density plays in a positive manner in the likelihood of a helminth infective form (egg or larva) coming into contact with a host, which contrasts with what happens in the case of no-FES cycles [104,105].

#### 4.2.2. Ageohelminth, Pseudogeohelminth and Geohelminth Cycles

Nematodes with an ageohelminth cycle (*S. stroma* and *S. frederici*) are, together, the most prevalent in both areas. This result is bound to be related to the ease with which transmission of these oxyurids, by means of direct contact between the host population and the habitat they share, occurs. However, the low prevalence observed during the first PFYs in the burned area could be due to the scarce wood mouse population at the beginning of the regeneration process. This hypothesis is corroborated by the fact that when the population density of the wood mouse increases in the burned area, the annual prevalence is generally higher than the global prevalence of the control area.

The fact that there were no clear correlations between climate variables and the host population density with prevalence is explicable by the usually high prevalence of oxyurids in both areas.

Pseudogeohelminths (*T. muris*, *E. bacillatus* and *A. tetraptera*) showed the lowest prevalence in both areas among the three types of monoxenous life cycles considered, likely to be due to the lower probability of coinciding with a viable embryonated egg in the shared space with respect to the other infective free environmental stages.

Although the embryonation of helminth eggs in the environment is known to be favoured by rainfall (more humid soil), the influence of this variable was detected in the burned area only, together with the influence of the PFP. This fact confirms, yet again, the higher vulnerability of the area in regeneration to the changes in the ecosystem along the seasonal periods. The burned area suffers the effects of climatic changes in a more profound manner as the ecosystem is in a permanent evolutionary process and is, therefore, more vulnerable to the climate conditions than the control area [15].

The influence, in both areas, of the host age might be related to the higher mobility of older *A. sylvaticus* individuals, increasing the exposure time and in turn the likelihood of being infected by a helminth [23], in this case ingesting an infective helminth egg.

The negative influence of certain environmental characteristics on *H. polygyrus* larvae, the only nematode with a geohelminth life cycle, is well known [27]. Among the factors with a negative influence are some climate variables and the diminishing pH of the soil [106], resulting in more extreme survival conditions for the larvae. However, the absence of correlation between the prevalence of *H. polygyrus* and climate variables or the host population density in the burned area is surprising. In the control area, a negative correlation between the prevalence and the minimum temperature reflects the direct influence of the temperature on the evolution of these free-living nematode larvae. In the burned area, factors other than climate-related ones, such as the loss of nutrients and the alkalinity of the soil as a consequence of the impact of the wildfire and the regeneration process, could exercise a more important influence, as reflected during the study of the 2nd–12th PFY period in which the importance of the NDVI vegetation index related to vegetal cover, which in turn depends on the pH of the soil and the presence of nutrients, was emphasized [16].

In both areas, the prevalence of geohelminths was influenced by the PFY and the host age. However, in other studies carried out previously on this post-fire regeneration process, the variability of the population of this geohelminth was observed, confirming the pronounced differences in favour of the control area during the more advanced regeneration periods [16].

### 4.3. Helminth Biodiversity

#### 4.3.1. Mean Species Richness

The annual mean species richness in the burned area is usually above 3, with the mean global value also being higher than the value of the control area. The values obtained in the burned area are very similar to those reported in other studies on the wood mouse in the Iberian Peninsula [39,42], being slightly higher than those reported in the burned area of previous periods analysed [14,17]. However, the values in the burned area are higher than those obtained in other wood mouse studies such as a long-term study carried out during 26 years in England, which reported an overall mean species richness of only 1.01 [26].

Among the influences exercised by the climate on the species richness, the positive correlation of mean temperature and rainy days in the burned area only stands out. The logical and predictable influence of the host age on the species richness has also been reported in other peninsular enclaves, such as the Erro river valley [42] and Sierra Espuña [39], and in England [26], as well as in the burned area, but also in the control area in previous periods analysed in Serra Calderona NP, i.e., in the 2nd–10th PFY period [17].

However, although in both areas the species richness seems to be influenced by the PFY and the host age, the influence of the PFP was observed in the burned area only. These results reflect, therefore, that, 18 years after the wildfire, the burned area is still undergoing a regeneration process and presents a higher instability of the helminth community of *A. sylvaticus* due to extrinsic factors such as climatology and annual seasonality, which influence the infracommunities of the burned area but not those of the control area.

#### 4.3.2. Helminth Diversity/Evenness—Brillouin Index

The generally low values of the specific diversity indices calculated, both the diversity/evenness and richness indices, indicate that there is a clear dominance of only a few helminth species belonging to the helminth community as a whole, in both areas. However, a higher richness and lower dominance in most of the analysed PFYs is observed in the burned area with respect to the control. Although some indices tend to be sensitive to the sample size, it is evident that the higher diversity of the wood mouse helminth community along the regeneration process in the burned area is less uniform than in the control area. It is important to note that during some of the sampling years, between the 5th and 17th PFY, the majority of the indices in the burned area were below those found in the control area. This is probably due to the higher incidence of the annual fluctuations in the burned area, as can be observed by the variation in Brillouin index values, the only index calculated at infracommunity level. Moreover, the nonexistence of significant differences between the PFY values of the diversity indices in both areas contrasts with the results obtained during the analysis of the 2nd–10th PFP [17]. The inclusion of eight more PFYs in the analysis could confirm the trend towards the similarity observed between both areas.

Surprisingly, the values of the diversity indices do not correlate with any of the climate variables or the population density of the wood mouse in either the burned or control areas. However, the interactions between the Brillouin index and the PFY and season could explain the annual fluctuations of its values. The relation of the wood mouse age with the Brillouin index values could be linked to the different diet and territorial behaviour of each subpopulation. Other studies carried out in the UK, Spain and Portugal reported a higher helminth diversity and species richness in the adult subpopulation [23,26,39,41]. This effect of the host age can be related to two facts: first, the increase in the host age goes together with the exposure to helminth infestations; second, the parasite burden accumulates along time [14,17,23,24,26,107,108].

The wood mouse population density could be responsible, among other factors, for the high prevalence values in the burned area, and the higher prevalence observed in this area with respect to the control area [105,109]. Moreover, the infracommunities, composed of up to nine species, are frequently larger than those of the control area, and those reported from other peninsular enclaves analysed, as well as those reported in previous studies carried out in Serra Calderona NP. These two characteristics of the helminth community of *A. sylvaticus* confirm the higher diversity of the area in regeneration.

The lower stability of the burned area goes together with a higher diversity of the helminth community, which may also be related to the post-fire succession in this Mediterranean forest ecosystem. The suppression of the host immune system may occur during this regeneration process as a result of the increase in helminth diversity, favouring the presence of concurrent species and, consequently, the increment in the species richness [17,110].

### 4.4. Biological Indicators

Previous studies carried out on the post-fire regeneration process in Serra Calderona NP [14,16,17] proposed certain aspects of the bioecology and the diversity of the helminth community of the wood mouse in the burned area as biological indicators of this process; some of which have been corroborated in the entirety of the 2nd–18th PFY period:

- the higher influence of the climate as well as seasonality (PFP) in the burned area on the prevalence of FES and pseudogeohelminth cycles have been reaffirmed as biological indicators of the higher vulnerability of the post-fire regeneration area to periodical changes in the ecosystem.
- Moreover, two other new biological indicators can be proposed:
- the higher mean species richness, the higher value of indices of specific diversity, the higher global prevalence and the presence of infracommunities made up of more helminth species in the burned area are proposed as biological indicators of the higher richness and biodiversity of the area in post-fire regeneration, and
- the influence of the PFP on the mean species richness and the annual fluctuations of the Brillouin index values in the burned area are proposed as biological indicators of the higher instability of the helminth community in areas in regeneration.

### 4.5. Limitations of the Study

The current research project on "the use of helminths of small mammals as biological indicators of the post-fire regeneration process in Mediterranean ecosystems", initiated almost three decades ago, had some limitations in its initial design due to the difficulties in obtaining the zoological material in the burned area of a natural park, considering both the great trapping effort needed as well as the obtention of dead animals to the helminthological study. Moreover, with the aim being to mitigate the low number of animals captured in the burned area, the two prospected areas affected by the wildfire (very close to each other and ecologically very similar) were considered throughout as a single burned area.

Furthermore, it should be taken into account that terrestrial ecosystems have, in general, not been as well studied as aquatic ones, in which the use of biological tags is much more common and analysed in depth, especially due to the difficulty of obtaining sufficient amounts of representative zoological material. This is one of the reasons why there are no previous hypotheses about the evolution in many terrestrial ecosystems, and that we are forced to talk, on many occasions, of trends and, therefore, more of biological indicators than biological tags.

Along the periods analysed (2nd–5th, 2nd–10th, 2nd–12th PFYs) the same extrinsic and intrinsic factors and dependent variables derived from the helminth community and the helminth component populations in both areas have been considered, providing new biological indicators and confirming, or not, those previously proposed, based, for example, on the differences concerning the behaviour of these helminth species and the population dynamics of the wood mouse in the burned area when compared to the control area. However, although this ecological analysis has been improved along the studied periods, including new statistical tests, and that, in our opinion, both the BLR and ANOVA showed their potential to follow the influence of extrinsic and intrinsic factors on the evolution/changes that have taken place in both areas, the inclusion of multivariate tests could lead to new findings that confirm the biological indicators of the post-fire regeneration process or even to the proposal of new ones. The analysis of new periods, currently in process, will incorporate the above-mentioned multivariate analysis.

## 5. Conclusions and Final Remarks

In general, 18 years after the wildfire that affected a large part of Serra Calderona NP, the post-fire regeneration process is still ongoing, as can be deduced from the majority of the results obtained and the biological indicators postulated.

The area in regeneration remains more vulnerable to seasonal and annual changes due to extrinsic factors such as climate conditions, vegetal recovery and sporadic but repetitive environmental impacts, mainly of anthropic nature.

The general comparative analysis between the burned and the control areas shows some differences, even after nearly two decades, such as:

- the burned area still hosts a wood mouse helminth community with two further species, *H. straminea* and *G. arfaai*;
- global prevalence as well as those of some helminth component populations in the burned area are still higher than those observed in the control area, although the trend towards their similarity seems to be accelerating;
- considering the bioecological level of the helminth community, FES-type cycles are still more prevalent in the burned area;
- the biodiversity, concerning species richness as well as helminth diversity, are still higher in the area in regeneration.

The observations and biological indicators that show the still existing differences between the two areas as a consequence of the post-fire regeneration process are directly related to some of the analysed factors, with the most outstanding being the climate variables related to the year and PFP before capture, among extrinsic factors and host age, and among intrinsic factors.

Once again, the important role that helminth parasites of the wood mouse play as biological indicators of the post-fire regeneration process in Mediterranean ecosystems has been demonstrated, making it possible to postulate that the analysis of the state of regeneration of these processes should take into account as a whole the aspects related to: the regeneration of the ecosystem, mainly concerning the succession and vegetation cover, the evolution and composition of the fauna, both invertebrates and vertebrates, and the climate conditions along the entire process; the host population analysed, the evolution of population dynamics and structure; the behaviour of the helminth community, the analysis of the annual evolution of the helminth component species and the influence of intrinsic and extrinsic factors on these.

## Figures and Tables

**Table 1:** Number of wood mice analysed and number of individuals captured per 100 trap-nights by post-fire year (PFY) in the burned (B) and control (C) areas.

	n° of *A. sylvaticus* Analysed		*A. sylvaticus* Captured per 100 Trap-Nights	
**PFY**	B	C	B	C
Second	21	11	2.20	1.82
Third	19	27	1.64	2.55
Fourth	61	8	11.19	2.39
Fifth	51	19	13.22	5.30
Sixth	107	50	16.00	13.09
Seventh	53	11	17.88	9.09
Eighth	26	11	8.94	4.85
Ninth	17	8	8.18	5.00
Tenth	53	11	16.21	5.15
Eleventh	49	25	35.68	25.45
Twelfth	17	12	19.74	16.82
Thirteenth	15	4	12.73	12.42
Fourteenth	33	7	18.18	8.79
Fifteenth	29	3	10.45	3.64
Sixteenth	39	9	20.91	11.52
Seventeenth	40	14	15.61	16.06
Eighteenth	45	12	18.64	8.18

**Table 2:** Distribution of wood mice analysed by host age and sex and season of capture in the burned (B) and control (C) areas.

		Host Age							
		Juveniles		Sub-Adults		Adults		Total	
Host Sex	Season	B	C	B	C	B	C	B	C
Males	Autumn	0	0	21	3	20	9	41	12
	Winter	17	19	102	21	63	23	182	63
	Spring	11	1	51	19	38	7	100	27
	Summer	4	0	22	7	43	21	69	28
	TOTAL	32	20	196	50	164	60	392	130
Females	Autumn	2	0	13	8	4	2	19	10
	Winter	25	20	65	24	41	17	131	61
	Spring	10	4	52	25	20	2	82	31
	Summer	9	1	31	8	11	1	51	10
	TOTAL	46	25	161	65	76	22	283	112

**Table 3:** Annual average of climate variables included in the study: temperature in °C, minimum (minT), medium (medT) and maximum (maxT); accumulated precipitation (accP) in tenths of mm; and rainy days (Rd); by post-fire year (PFY) in the burned (B) and the control (C) areas; data not recorded by the climate stations considered (nr).

	minT		medT		maxT		accP		Rd	
PFY	B	C	B	C	B	C	B	C	B	C
**1st**	9.9	9.8	15.9	16.0	21.9	22.2	3791	4725	71	109
**2nd**	10.6	10.1	16.7	16.6	22.7	23.1	3715	3987	53	98
**3rd**	11.3	10.2	16.8	16.6	22.3	23.0	2523	2524	56	100
**4th**	11.1	10.3	16.4	16.5	21.8	22.6	5086	6262	88	134
**5th**	10.7	10.3	15.7	16.5	21.6	22.7	4700	4901	84	126
**6th**	10.5	10.8	15.6	17.1	20.7	23.3	3292	3774	65	116
**7th**	10.0	10.6	15.7	17.0	21.4	23.3	3410	3679	51	93
**8th**	11.0	10.3	16.9	16.6	22.9	22.9	2370	2537	48	92
**9th**	10.3	11.6	16.6	17.5	22.5	23.4	6068	6814	52	107
**10th**	9.6	10.7	13.8	16.4	20.2	22.1	6280	6167	21	117
**11th**	nr	nr	17.4	17.1	nr	nr	5532	4467	93	111
**12th**	nr	nr	16.9	16.4	nr	nr	5373	7542	105	134
**13th**	nr	nr	16.6	15.7	nr	nr	3621	3020	73	99
**14th**	nr	nr	17.2	16.3	nr	nr	4980	5476	89	99
**15th**	5.9	7.2	17.3	18.2	29.4	28.4	4698	4929	73	81
**16th**	5.4	7.2	16.4	17.8	28.4	28.6	5074	5477	68	62
**17th**	6.2	7.4	16.5	17.4	28.3	28.3	5598	5951	67	59
**18th**	5.6	7.2	16.6	17.6	29.1	29.7	4516	4025	83	61

**Table 4:** Selected characteristics of the helminth community of 917 wood mice analysed from Serra Calderona Natural Park.

Helminth Species	Site	Monox.	Heterox.	FES	No-FES	Other Characteristics
TREMATODA						
*Brachylaima* spp.	SI		X		X	Infective form in a terrestrial snail
CESTODA						
*Taenia parva* larvae	BC		X	X		Wood mice act as intermediate host
*Taenia martis* larvae	BC		X	X		Wood mice act as intermediate host
*Mesocestoides* spp. larvae	BC		X		X	Wood mice act as 2nd intermediate host
*Pseudocatenotaenia matovi*	SI		X		X	Infective form in a terrestrial arthropod
*Skrjabinotaenia lobata*	SI		X		X	Infective form in a terrestrial arthropod
Catenotaeniidae Gen. spp.	SI		X		X	Infective form in a terrestrial arthropod
*Hymenolepis straminea*	SI		X		X	Infective form in a terrestrial arthropod
*Gallegoides arfaai*	SI		X		X	Infective form in a terrestrial arthropod
NEMATODA						
*Trichuris muris*	C	X		X		pseudogeohelminth
*Eucoleus bacillatus*	SW	X		X		pseudogeohelminth
*Aonchotheca annulosa*	SI		X		X	Infective form in a terrestrial invertebrate
*Heligmosomoides polygyrus*	SI	X		X		geohelminth
*Syphacia stroma*	SI	X		X		ageohelminth
*Syphacia frederici*	LI/C	X		X		ageohelminth
*Aspiculuris tetraptera*	SI	X		X		pseudogeohelminth
*Mastophorus muris*	S		X		X	Infective form in a terrestrial arthropod
Nematoda Gen. spp. larvae	SI		X	?	?	Stray parasitism/mice as paratenic host

SI, small intestine; BC, body cavity; C, caecum; SW, stomach wall; LI, large intestine; Monox., monoxenous or direct life cycle; Heterox., heteroxenous or indirect life cycle.

**Table 5:** Characterization of the helminth community of the wood mouse in the burned (*n* = 675) and control (*n* = 242) areas. CI, confidence interval; SE, standard error. FES, helminths which have a free-environmental infectious stage for the wood mouse.

Helminth Species	Prevalence (95%CI)		Mean Abundance (SE)		Median Intensity (Range)	
	Burned	Control	Burned	Control	Burned	Control
*Brachylaima* spp.	3 (2–5)	0. 5 (0–1)	0.13 (0.05)	0.004 (0.004)	4.30 (1–20)	1.00 (1)
*Taenia parva* larvae	6 (4–8)	8 (5–12)	0.08 (0.01)	0.16 (0.04)	1.24 (1–3)	1.95 (1–6)
*Taenia martis* larvae	3 (2–5)	3 (1–6)	0.04 (0.01)	0.04 (0.02)	1.26 (1–3)	1.25 (1–3)
*Mesocestoides* spp. larvae	1 (0.4–2)	0.5 (0–1)	0.26 (0.20)	0.21 (0.21)	25.29 (3–136)	52.00 (52)
*Pseudocatenotaenia matovi*	7 (5–9)	10 (7–14)	0.27 (0.08)	0.25 (0.08)	3.68 (1–33)	2.61 (1–15)
*Skrjabinotaenia lobata*	11 (9–14)	4 (2–7)	0.71 (0.16)	0.07 (0.03)	6.22 (1–83)	1.80 (1–5)
Catenotaeniidae Gen. spp.	5 (4–7)	4 (2–7)	0.34 (0.13)	0.09 (0.03)	6.24 (1–82)	2.10 (1–5)
*Hymenolepis straminea*	0.5 (0–1)	–	0.01 (0.005)	-	2.0 (1–3)	–
*Gallegoides arfaai*	7 (5–9)	–	0.11 (0.02)	-	1.65 (1–4)	–
*Trichuris muris*	21 (18–24)	7 (4–11)	0.51 (0.05)	0.14 (0.07)	2.37 (1–16)	2.06 (1–16)
*Eucoleus bacillatus*	16 (14–19)	8 (5–12)	1.17 (0.30)	0.25 (0.07)	7.42 (1–171)	3.21 (1–9)
*Aonchotheca annulosa*	25 (22–28)	11 (7–16)	3.95 (0.82)	0.58 (0.18)	15.98 (1–291)	5.38 (1–27)
*Heligmosomoides polygyrus*	13 (11–16)	36 (30–42)	1.00 (0.28)	2.45 (0.45)	7.70 (1–113)	6.82 (1–65)
*Syphacia stroma*	51 (47–55)	20 (15–26)	50.95 (6.37)	6.82 (1.77)	99.10 (1–1937)	34.38 (1–212)
*Syphacia frederici*	20 (17–23)	28 (23–34)	26.06 (6.05)	28.94 (12.07)	122.44 (1–2646)	104.54 (1–2846)
*Aspiculuris tetraptera*	2 (1–3)	3 (1–6)	0.13 (0.07)	0.69 (0.63)	7.42 (1–41)	23.86 (1–52)
*Mastophorus muris*	11 (9–14)	17 (13–22)	0.36 (0.07)	0.35 (0.08)	3.23 (1–35)	2.00 (1–14)
Nematoda Gen. spp. larvae	0.1 (0–0.5)	0.5 (0–1)	0.002 (0.002)	0.004 (0.004)	1.00 (1)	1.00 (1)

**Table 6:** Total prevalence (P) of the five types of life cycles in the wood mouse, and parameters of statistically significant differences between them, in the burned (B) and control (C) areas.

Type of Life Cycles	Burned		Control			
	*n*	*P (%)*	*n*	*P (%)*	*χ* ^2^	*P*
FES	540	80.00	162	66.94	16.203	<0.0001
No-FES	323	47.85	86	35.54	10.441	0.0012
Ageohelminths	446	66.07	102	42.15	41.415	<0.0001
Pseudogeohelminths	243	36.00	34	14.05	39.678	<0.0001
Geogeohelminths	88	13.04	87	35.95	59.092	<0.0001

FES, helminths which have a free-environmental infectious stage for the wood mouse.

**Table 7:** Diversity characteristics of the helminth community of the wood mouse in the burned and control areas.

Diversity/Uniformity Index	Burned	Control
Shannon index	1.11	1.05
Simpson index	0.55	0.47
Berger–Parker index	0.39	0.30
Shannon evenness index	0.38	0.38

**Table 8:** Biodiversity characteristics of the helminth community of the wood mouse in the burned and control areas.

Biodiversity Characteristics		Burned	Control
Mean species richness	X	2.03	1.60
	SE	0.06	0.09
Brillouin index	X	0.23	0.18
	SE	0.01	0.02
	Max.	3.74	1.39
BI infected *A.s.* only	X	0.26	0.23
	SE	0.02	0.02
% of *A.s.* infected		87.70	77.69

**Table 9:** Values of correlations between the prevalence of the different kinds of life cycles and values of species richness and the Brillouin index with climate data and host population density (values of the year prior to capture) by means of the Spearman correlation coefficient (Rho) and the associated P values in parentheses.

	Host Density		Min. Temp.		Mean Temp.		Precipitation		Rainy Days	
Bioecology	Burned	Control	Burned	Control	Burned	Control	Burned	Control	Burned	Control
FES	-	-	-	−0.564 (0.045)	-	-	-	-	0.709 (0.001)	-
No-FES	-	−0.626 (0.009)	-	-	-	-	-	−0.691 (0.002)	-	-
Ageohelminths	-	-	-	-	-	-	-	-	0.499 (0.041)	-
Pseudogeohelminths	-	-	-	-	-	-	-	-	0.581 (0.014)	-
Geohelminths	-	-	-	−0.805 (0.001)	-	-	-	-	-	-
**Biodiversity**										
Species richness	-	-	-	-	0.517 (0.034)	-	-	-	0.486 (0.048)	-

FES, helminths which have a free-environmental infectious stage for the wood mouse.

**Table 10:** Logistic regression models for prevalence of the five types of life cycles by year and period of capture, host age and host sex, in the burned and control areas, expressed by *χ*^2^ values with associated probabilities (*P*) for the model created including independent variables. *df* = degree of freedom.

		Burned		Control	
Independent Variables in the Model	df	*χ* ^2^	*P*	*χ* ^2^	*P*
**FES**					
Post-fire period	34	101.502	0.0001	-	-
Post-fire year	16	-	-	58.879	0.0001
**no-FES**					
Post-fire period/Host age	36	168.832	0.0001	87.933	0.0001
**Ageohelminths**					
Post-fire period	34	133.381	0.0001	86.277	0.0001
**Pseudogeohelminths**					
Post-fire period/Host age	36	205.873	0.0001	-	-
Post-fire year/Host age	18	-	-	58.866	0.0001
**Geohelminths**					
Post-fire year/Host age	18	94.549	0.0001	115.967	0.0001

FES, helminths which have a free-environmental infectious stage for the wood mouse.

**Table 11:** ANOVA models for values of species richness and the Brillouin index of the wood mouse by year and period of capture, host age and host sex, in the burned and control areas, expressed by F values with associated probabilities (*P*) for the model created including independent variables. *df* = degree of freedom.

		Burned		Control	
Independent Variables in the Model	df	*F*	*P*	*F*	*P*
**Species richness**					
Post-fire year	16	5.060	0.0001	2.924	0.0001
Host age	2	23.705	0.0001	6.791	0.002
Post-fire period	30	2.805	0.0001	-	-
Year of capture/Host sex	16	1.687	0.046	-	-
Post-fire period/Host sex	22	2.290	0.001	-	-
Post-fire period/Host age	31	1.507	0.042	-	-
**Brillouin index**					
Post-fire year	16	1.749	0.0.035	2.449	0.003
Host age	2	12.972	0.0001	8.507	0.0001
Post-fire period	30	2.082	0.001	1.873	0.014
Host sex/Host age	2	-	-	3.390	0.037
Post-fire period/Host sex	22	1.575	0.048	-	-
